# Reliability and Validity of the Malay Mindful Eating Questionnaire (MEQ-M) among Overweight and Obese Adults

**DOI:** 10.3390/ijerph18031021

**Published:** 2021-01-24

**Authors:** Siti Munirah Abdul Basir, Zahara Abdul Manaf, Mahadir Ahmad, Nor Ba’yah Abdul Kadir, Wan Nur Khairunnisa Ismail, Arimi Fitri Mat Ludin, Suzana Shahar

**Affiliations:** 1 Dietetic Program and Centre for Healthy Aging and Wellness, Faculty of Health Sciences, Universiti Kebangsaan Malaysia, Jalan Raja Muda Aziz, Kuala Lumpur 50300, Malaysia; sitimunirah.abdulbasir@gmail.com (S.M.A.B.); suzana.shahar@ukm.edu.my (S.S.); 2 Clinical Psychology & Behavioural Health Program, Center for Community Health Studies (ReACH), Faculty of Health Sciences, Universiti Kebangsaan Malaysia, Jalan Raja Muda Aziz, Kuala Lumpur 50300, Malaysia; mahadir@ukm.edu.my; 3 Centre for Research in Psychology and Human Well-being, Faculty of Social Sciences and Humanities, Universiti Kebangsaan Malaysia, Selangor, Bangi 43600, Malaysia; aknbayah@ukm.edu.my (N.B.A.K.); nisaismail281@gmail.com (W.N.K.I.); 4 Biomedical Science Program and Centre for Healthy Aging and Wellness, Faculty of Health Sciences, Universiti Kebangsaan Malaysia, Jalan Raja Muda Aziz, Kuala Lumpur 50300, Malaysia; arimifitri@ukm.edu.my

**Keywords:** mindfulness, obesity, MEQ, reliability, validity

## Abstract

The Mindful Eating Questionnaire is a reliable tool for the assessment of mindful eating behavior among the general population. This study aimed to determine the reliability and validity of The Malay Mindful Eating Questionnaire (MEQ-M) in a sample of overweight and obese adults. This is a cross-sectional survey which involved 144 overweight and obese adults in a selected public university. After linguistic validation of the Malay version of the MEQ, exploratory factor analysis (EFA) with varimax rotation was performed on the scale constructs. The psychometric properties of the MEQ were assessed through Cronbach’s alpha and intraclass correlation coefficient (ICC) analysis. The EFA of the MEQ produced a seven-dimensional model (58.8% of overall variances). The concurrent validity analysis between total MEQ scores and total Mindfulness Attention Awareness Scale (MAAS) scores indicated a weak non-significant correlation (*p* = 0.679). The internal consistency reliability of the MEQ was reasonable (Cronbach’s α = 0.64). The agreement stability of the MEQ over eight weeks was poor (ICC = 0.10). In conclusion, the psychometric properties of the Malay-translated MEQ are acceptable through construct validity and internal consistency reliability tests. This instrument may be used for assessing mindful eating habits in the Malaysian population, especially among overweight and obese adults.

## 1. Introduction

Mindfulness is commonly understood as the ability of being open, accepting, and present in the moment [1]. Mindfulness trainings such as Mindfulness-Based Stress Reduction (MBSR), Mindfulness-Based Cognitive Therapy (MBCT), Acceptance and Commitment Therapy (ACT), and Dialectical Behavioral Therapy (DBT) are often described as interventions that focus to promote non-judgmental and moment-to-moment awareness of the present experience. The role of mindfulness in encouraging behaviors related to healthy body weight maintenance and reducing overweight and obesity has gained attention among scholars [2]. This practice has been beneficial to control food cravings, portion size, body mass index, and body weight [3]. Mindful eating refers to being conscious of physical sensation and emotion while eating or in a food-related environment [1]. It helps in improving one’s sensitivity to the physical cues of hunger, satiety, eating speed, and the food atmosphere [3]. These cues are crucial to self-regulate one’s desire to consume high-calorie foods. Studies have shown that mindful eating helps in reducing negative eating behaviors, sweets consumption, and serving sizes of energy-dense foods [4,5,6].

There are many factors associated with eating behaviors, such as physiological (i.e., chronotype), social (i.e., coworker influence), environmental, and psychological (i.e., stress, mood) [7,8,9]. It has been hypothesized that obesity-related eating behaviors are partially associated with the inability to identify and respond to internal cues of hunger and satiety [10,11] (pp. 75–91), [12] (pp. 913–933). This lack of response to internal cues is correlated with increased overeating episodes and a greater risk for weight gain [13,14,15]. In addition, emotional dysregulation has been associated with emotional and stress eating [16,17,18,19]. Studies have shown that compulsive overeating and higher preference for high calorie, fat, sugar, and/or high sodium foods are the result of negative emotions and acute stress [20,21,22]. Emotional eating has been shown to be a strong indicator of obesity and is negatively associated with weight loss and its maintenance [23,24,25]. Furthermore, a restricted diet and increased physical activity can result in physiological discomforts that may impose an added barrier to long-term weight loss [24].

There are several tools available in measuring eating behaviors such as Night Eating Syndrome Questionnaire (NEQ), Three-Factor Eating Questionnaire (TFEQ) and Binge Eating Scale (BES). The Mindful Eating Questionnaire (MEQ) is the first scale developed by Framson and colleagues that measures mindful eating [1]. It is a 28-item self-report instrument that consists of five mindful eating domains: awareness, disinhibition, distraction, emotional response, and external cues. This questionnaire has been validated previously among healthy adults aged 18 to 80 years old. The study showed good internal consistency with a reliability of 0.64 for the MEQ score [1]. In addition, each subscale had internal consistency ranging from 0.64 to 0.83. It was also reported that there were modest (0.14) to moderate (0.47) correlation among all subscales with the exception of correlation between external cues and emotional response. Another validation study among overweight and obese pregnant women yielded the same five domains of the MEQ [26]. It was found that the MEQ has poor internal consistency reliability of the summary score (0.56). As for the subscales, only the external cues subscale was not internally consistent with Cronbach’s alpha of 0.31. Its reliability was further supported by test-retest analysis, where the total and subscale scores were ranged between 0.62 to 0.85. To add, positive correlations were also observed between the MEQ subscales and the Mindful Attention Awareness Scale (MAAS) [26]. Another study of the Persian version of MEQ among women seeking weight loss reported satisfactory internal consistency for the total score and the subscales (0.73–0.81) and satisfactory test-retest reliability ranging from 0.73 to 0.91. [27]. Its construct validity analysis resulted in five domains which were similar to the original study. Contrary, the Italian version of the MEQ resulted in a 20-item pool where only two domains emerged; awareness and recognition [28]. Clementi et al. also found that both domains have satisfactory internal and test-retest reliability, and were associated with general mindfulness. Meanwhile, the MAAS is a standard tool used to assess mindfulness in everyday life among the general population [29]. It consists of a 15-item self-reported single-factor scale that focused on the mindfulness construct’s attention awareness component.

The prevalence of overweight and obesity had significantly increased between 1976 and 2016 globally, in which half of them (52%) were adults over 18 years of age [30]. In Malaysia, the National Health and Morbidity Survey (NHMS) reported that the prevalence of overweight and obesity among adults increased by 5% from 2011 to 2015 [31,32]. Moreover, the prevalence by age in 2015 showed an increasing trend from 34.8% among 18–29 years to 60.2% among 50–59 years. Obesity is associated with increased risk of many non-communicable diseases (NCDs), including diabetes, cardiovascular disease, depression, some cancers, and respiratory disease [33,34,35,36,37]. Moreover, it negatively impacts bone health, quality of life, and functional capacity [38,39,40]. Consequently, obesity is also associated with expensive health care costs [41,42,43]. One study suggested that obese adults have difficulty in reflecting on the impact of obesity on their social and relational functioning despite having psychological difficulties [44]. Considering the increasing trend in obesity prevalence in Malaysia, a locally validated instrument is essential in conducting research and intervention activities. To the best of our knowledge, no measure has been carried out to assess mindful eating behavior in Malaysia’s overweight and obese adults. Moreover, there is currently no instrument measuring mindful eating (in general) in the Malaysian context using its local language. Several studies have shown that eating mindfully was associated with a lower BMI [45,46] and reduced body weight [47]. Thus, the objective of our present study was to determine the reliability and validity of the MEQ-M in a sample of overweight and obese adults. This is the first study to examine the reliability and validity of the Mindful Eating Questionnaire (MEQ) among this population. We hypothesized that the MEQ would have similar results to a previous study [40] where the questionnaire would be valid and reliable among overweight and obese Malaysian adults. As mindful eating was generally associated with general mindfulness in previous studies, we hypothesized that the MEQ-M total score would be positively correlated with the Mindful Attention Awareness Scale (MAAS).

## 2. Materials and Methods

### 2.1. The Questionnaire

The MEQ contains five subscales: awareness (seven items), distraction (three items), disinhibition (eight items), emotional responses (four items), and external cues (six items). The eating behaviors are rated on a four-point Likert scale; 1—never/rarely, 2—sometimes, 3—often, and 4—usually/always. Reverse scoring was applied to questions 1, 2, 6, 7, 9, 11, 17, 18, 19, 27, and 28.

### 2.2. Translating the Questionnaire

A back-translation method was used to create the Malay-translated version of the MEQ [48]. The original version of the MEQ was first translated into the Malay language by two authors who are bilingual (English and Malay). The translated version was then piloted to 10 university staff members to test for clarity. Some unclear terms and phrases were noted. The questionnaire was then carefully checked for clarity, accuracy, the language’s suitability, and linguistic errors by two independent researchers. Once clarity and accuracy had been established, the questionnaire was then back-translated from the Malay version to English by an independent translator. The revised and modified translated items are available in the Appendix A.

### 2.3. Data Collection

This study was a cross-sectional survey to assess the psychometric properties of the Malay version of MEQ conducted on 144 overweight and obese working adults conveniently recruited in a selected public university. These participants were recruited by our researcher from a health screening program held by the university among the staff. The sample size was determined based on a 5:1 ratio, where the sample size is expected to be a least five times the total number of items in the questionnaire [49] (pp. 86–99). Respondents were eligible if they met each of the following criteria: BMI ≥ 25.0 kg/m^2^, age 18–59 years, and no chronic diseases, such as cancer, kidney diseases, or heart diseases. Exclusion criteria were pregnant and/or breastfeeding women, having any severe mood disorder controlled by pharmaceuticals, and the use of pharmaceutical weight control. The socio-demographic information of the participants’ age, gender, educational level, monthly household income, and types of work were collected through a questionnaire. The BMI of the participants were measured using the TANITA Body Composition Analyzer (model TBF 300, Tanita Corporation, Tokyo, Japan). The Malay-translated MEQ was distributed via a Google Form. Ethical approval was obtained before data collection from the Universiti Kebangsaan Malaysia Medical Research Ethics Committee (UKM PPI.800-1/1/5/JEP-2019-391). Respondents were briefed on the purpose of the study and written consent was obtained.

### 2.4. Validation of MEQ-M

#### 2.4.1. Construct Validity

**Figure 1:**
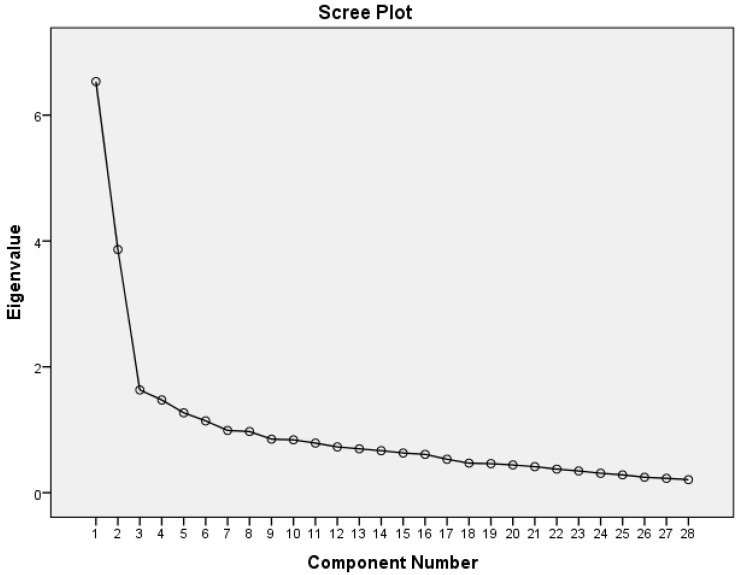
Scree plot of the eigenvalues for the principal component analysis of the Mindful Eating Questionnaire (MEQ).

Factor analysis enables the determination of the underlying subdomains of a questionnaire [50]. Exploratory factor analysis (EFA) was recommended for establishing equivalence and factor structure validation of the translated and adapted questionnaires performed in different sample populations [51]. In this study, the MEQ factor structure was determined by using principal component analysis with varimax rotation [52]. This rotation produces a simpler solution and uncomplicated interpretation while maximizing the total variances of the squared loadings correlation between variables and factors. An eigenvalue of >1, a factor loading of ≥0.4 and a scree plot were applied for this study (Figure 1) [26].

#### 2.4.2. Concurrent Validity

The Mindful Attention Awareness Scale (MAAS) is a 16-item tool that measures the frequency of mindfulness in daily life using general and situation-specific questions [53]. This instrument uses a six-point Likert scale from 1 (almost always) to 6 (almost never), in which the mean score can range from 1 to 6. Higher MAAS scores indicate greater mindfulness. This questionnaire was translated into the Malay language and validated by Zainal and colleagues [29].

#### 2.4.3. Reliability: Internal Consistency

The MEQ’s internal consistency or homogeneity was assessed using the coefficient Cronbach’s alpha and McDonald’s omega (range 0–1). A coefficient value of ≥0.70 indicates a satisfactory internal consistency [54]. In addition, the internal consistency of the MAAS was carried out.

#### 2.4.4. Reliability: Test-Retest

The objective of this analysis is to measure an instrument’s or test’s stability over time. This is performed by administering the same test to the same subject at two different points. For this study, the intraclass correlation coefficient (ICC) was used to estimate the reliability of the scale. The interpretation of the agreement levels by ICC are as follows: 0.0–0.2 as small, 0.21–0.40 as fair, 0.41–0.60 as moderate, 0.61–0.80 as substantial, and 0.81–1.0 as almost perfect [55]. Statistical analysis was performed using Statistical Package for the Social Sciences 25.0 (SPSS, Inc., Chicago, IL, USA), in which significance was set at *p*-values < 0.05. All variables were tested for normality using the Kolmogorov–Smirnov, skewness, and kurtosis analysis. Since our data were normally distributed, Pearson’s correlation test was employed. Participants with incomplete responses of the MEQ-M and MAAS were excluded from the analysis.

## 3. Results

### 3.1. Participants

In total, 144 participants (41 males, 103 females) were included in this study. The mean age of the participants was 40.3 ± 6.9 years. The socio-demographic of the participants are presented in Table 1.

### 3.2. Construct Validity

The MEQ was analyzed by principal component factor analysis with varimax rotation. The overall Kaiser-Meyer-Olkin measure of sampling adequacy was 0.804. Bartlett’s test for sphericity produced a significant result (*p* < 0.001), indicating that the variables were correlated with one another. Thus, our preliminary analyses confirmed the appropriateness of principal component factor analysis for the data. The Malay-translated questionnaire had seven factors. The percentage of variances explained by rotated factor matrices ranged from 4 to 14% per factor, with seven factors explaining 58.8% of the overall variance. Percentages refer to the variance explained by each factor as follows: Factor 1, 14.5%; Factor 2, 12.0%; Factor 3, 8.5%; Factor 4, 8.1%; Factor 5, 6%; Factor 6, 5.4%; and Factor 7, 4.5%. All items loaded 0.40 or above (Table 2).

### 3.3. Concurrent Validity

The concurrent validity of the Malay-translated MEQ was calculated using Pearson’s correlations with MAAS. The correlational analysis results between total MEQ-M scores and total MAAS scores indicated a weak non-significant correlation (*p* = 0.679). Only factors 1, 2, and 7 were significantly correlated with MAAS (Table 3). Factor 1, 2, 4, and 5 were positively correlated with MAAS, whereas factors 3, 6, and 7 were negatively correlated with MAAS. The MEQ subscale that showed the highest correlations with MAAS measures was factor 2.

### 3.4. Reliability

Cronbach’s alpha for the MEQ was 0.64, which indicates reasonable reliability. In contrast, the omega from McDonald’s reliability test was lower. Further analysis was carried out by eliminating the External Cues subscales, which increased the Cronbach’s alpha and McDonald’s omega reliability value to 0.71 and 0.61, respectively. As for the subscales, Cronbach’s alpha values ranged from 0.27 to 0.70, whereas McDonald’s omega values ranged from 0.58 to 0.80. The reliability coefficient for the subscales were similar from both tests, except for Factor 1 and Factor 2, which showed higher reliability from McDonald’s omega test. With the elimination of item 3 from the Factor 1 subscale, the alpha value increases to 0.78 from 0.52. Other than that, the elimination of item 26 resulted in improving the Factor 2 subscale from 0.616 to 0.683. The exclusion of item 14 from the Factor 5 subscale improves the alpha value from 0.54 to 0.67. As for the test-retest reliability, the ICC for the summary score of the MEQ-M was 0.104, which means that the items have a small agreement with each other. The ICC for each subscale is presented in Table 4. As for the MAAS, the Cronbach’s alpha was 0.88, which showed satisfactory internal consistency. The test-retest reliability coefficient was 0.295, which indicates a fair agreement between the scores.

### 3.5. Correlation between Sociodemographic Characteristics and the MEQ-M

Table 5 shows the correlation between sociodemographic characteristics and the total score of the MEQ-M and its subscales. Overall, there is no significant association observed with the exception to the Environmental Disinhibition subscale with BMI and gender (*p* = 0.013 and *p* = 0.044, respectively). Other than that, significant association was observed between age and two subscales (Environmental disinhibition and Emotional response) with *p*-values = 0.002 and 0.006, respectively. Furthermore, the questionnaire has poor correlation with all sociodemographic characteristics.

## 4. Discussion

The purpose of this study was to analyze the psychometric properties of the MEQ in overweight and obese Malaysian adult samples. This paper reports the translation procedure, validity, and reliability of the Malay-translated MEQ.

Construct validity (EFA) results were inconsistent with findings reported in previous studies [1,27]. These studies found that the 28-item MEQ had a good fit for a five-dimensional factor structure. On the contrary, we found a seven-dimensional model from the exploration. The seven factors were labeled as Factor 1 (environment disinhibition), Factor 2 (emotional response), Factor 3 (taste awareness), Factor 4 (emotion awareness), Factor 5 (portion disinhibition), Factor 6 (external cues of food), and Factor 7 (external cues of place). Each factor was loaded strongly with factor loadings from 0.43 to 0.79. Items 7, 13, and 26 had multiple cross-loading. If a more stringent factor loading was used at the cut-off point of 0.5 (25% shared variances), items 4 and 27 of the MEQ would be dropped and, it would become a 26-item scale. This yielded a six-dimensional model for the Malay-translated MEQ. A possible reason that this study produced a seven-dimensional model, in contrast to other studies, is due to cultural differences. Our population study might have had a different understanding of mindfulness. Studies have shown that cognitive and reasoning styles are different across cultures and may affect how questions are viewed and answered [56,57]. Language and cross-cultural variations may influence the respondents’ responses and affect the analysis of the questionnaire’s psychometric properties [58].

The Cronbach’s alpha and McDonald’s omega analysis showed that the Malay-translated MEQ was a reliable tool to be used among this group. The internal consistencies of the MEQ-M factors ranged from 0.54 to 0.70 for Cronbach’s alpha and 0.58 to 0.80 for McDonald’s omega, except for the External Cues subscales. This indicates that each factor’s items are moderately cohesive with each other in measuring specific mindful eating behavior. The low internal consistency of Factor 6 may be due to the small number of items. The internal consistency of Factor 7 could not be tested as there was only one item (Item 8) extracted. When item 8 was analyzed together with items 4 and 23 from Factor 6, the Cronbach’s alpha produced was 0.441. Exclusion of item 4, 8, and 23 (External Cues subscales) from the analysis has improved the reliability of the MEQ-M. Similarly, the external cues subscale was found to be invalid in a study among overweight and obese pregnant women [26]. The MEQ-M summary scale was 0.64, which is comparable to that reported by Abbaspoor (0.66) and Framson (0.64). In contrast, our findings showed better reliability compared to Apolzan and colleagues [26].

The test-retest reliability of the MEQ with an eight-week interval showed only a small agreement between the items. Three subscales (environmental disinhibition, emotional response, and taste awareness) achieved a fair correlation (0.26 to 0.45). In contrast, emotional awareness, portion disinhibition, external cues of food, and external cues of place subscales had a small correlation coefficient. We also found that portion disinhibition and external cues subscales had a negative ICC value due to a negative average covariance among items. This may have affected the overall ICC of the MEQ (0.10). A small ICC means that the items’ measurement was not stable over time. The Iranian version of the MEQ test-retest reliability had a high level of correlation, where all subscales’ ICC was ≥0.7 [27]. This discrepancy may be due to the different sample populations tested only among women with normal BMI. The ICC could be improved if a sizeable heterogeneous sample in terms of BMI was included. Moreover, they retested the questionnaire in a shorter period (four weeks) compared to the present study.

In correlation to MAAS, the MEQ correlated positively except for Factor 3, 6, and 7. However, the correlation was weak. This may suggest that individuals with higher mindfulness may or may not tend to eat mindfully. In contrast, MEQ was significantly correlated with MAAS among pregnant women [26]. The differences may be due to the new factors or domains produced from our study compared to the original MEQ which consists of only five factors. Other than that, the MAAS questionnaire focused exclusively on the attention awareness component of the mindfulness construct, whereas the MEQ includes constructs of eating behaviors and mindfulness. In our cultural context, this may reflect that these measures are not related to each other. Our study also found that there was no significant correlation between BMI and the MEQ-M’s subscales, except for the inverse relationship with the Environmental disinhibition subscale. This suggests that higher BMI was correlated with lower ability to stop eating even though they already feel full. Similarly, the subscales of the Iranian version of the MEQ also showed no correlation with BMI apart from the Awareness subscale [27]. In contrast, Framson et al. found that the overall scores of the MEQ was negatively associate with BMI [1].

This study was the first to establish the validity and reliability of the MEQ-M among overweight and obese healthy working adults. Since this study was conducted among overweight and obese adults, this limits the generalizability of the results to the general population. Furthermore, this instrument was a self-reported questionnaire. The respondents might have had difficulties with being aware of their eating experience when that questionnaire was given. They might have provided answers describing what they should do instead. In addition, there was no concurrent validation of the MEQ-M with other eating psychopathology, such as the binge eating scale and three-factor eating questionnaire, which may provide additional support for MEQ-M’s validation.

## 5. Conclusions

The study findings showed that the psychometric properties of the Malay-translated MEQ are acceptable through construct and internal consistency reliability. This instrument may be used for assessing mindful eating habits in the Malaysian population, especially among overweight and obese employees. However, the measurement by domains of the MEQ-M should be considered cautiously as it differs from the original domains. As the Malay-translated MEQ was only tested among overweight and obese university employees, we suggest subsequent studies among employees with normal BMI to further test its validity and reliability, especially with regards to its stability over time.

## Figures and Tables

**Table 1:** Characteristics of the study population, *n* = 144.

Age (years), mean ± SD	40.3 ± 6.9
BMI (kg/m^2^), mean ± SD (range)	31.7 ± 6.1 (25.0–60.6)
Gender, *n* (%)	
Male	41 (28.5)
Female	103 (71.5)
Education level, *n* (%)	
Secondary school	30 (20.8)
Tertiary	113 (78.5)
Others	1 (0.7)
Marital status, *n* (%)	
Single	12 (8.3)
Married	128 (88.9)
Divorced	4 (2.8)
Monthly household income (RM), *n* (%)	
≤RM 3000 (≤$718.99)	21 (14.6)
RM 3001-RM 5000 ($719.23–$1198.32)	50 (34.7)
RM 5001-RM 7000 ($1198.56–$1677.65)	28 (19.4)
>RM 7000 (>$1677.65)	45 (31.3)

**Table 2:** Factor loadings from the Malay Mindful Eating Questionnaire (MEQ-M) principal component analysis.

MEQ Items	Factor 1	Factor 2	Factor 3	Factor 4	Factor 5	Factor 6	Factor 7
Item 1	0.578						
Item 2	0.680						
Item 3	−0.786						
Item 9	0.726						
Item 11	0.690						
Item 13	0.483						
Item 18	0.654						
Item 6		0.595					
Item 7		0.556					
Item 17		0.646					
Item 19		0.580					
Item 27		0.558					
Item 28		0.745					
Item 10			0.450				
Item 12			0.690				
Item 15			0.732				
Item 21			0.492				
Item 26			0.429				
Item 20				0.736			
Item 22				0.425			
Item 24				0.593			
Item 25				0.736			
Item 5					0.717		
Item 14					0.597		
Item 16					0.556		
Item 4						0.757	
Item 23						0.657	
Item 8							0.765

**Table 3:** Correlations between MEQ-M and Mindfulness Attention Awareness Scale (MAAS).

MEQ-M Subscales	Factor 1 (Environmental Disinhibition)	Factor 2 (Emotional Response)	Factor 3 (Taste Awareness)	Factor 4 (Emotion Awareness)	Factor 5 (Portion Disinhibition)	Factor 6 (External Cues of Foods)	Factor 7 (External Cues of Place)
MAAS	0.295 ^b^	0.329 ^b^	−0.039	0.047	0.031	−0.147	−0.179 ^a^
Factor 1		0.592 ^b^	−0.161	0.105	0.092	−0.414 ^b^	−0.336 ^b^
Factor 2			−0.322 ^b^	−0.013	−0.06	−0.520 ^b^	−0.414 ^b^
Factor 3				0.447 ^b^	0.398 ^b^	0.396 ^b^	0.307 ^b^
Factor 4					0.449 ^b^	0.262 ^b^	0.078
Factor 5						0.114	0.000
Factor 6							0.359 ^b^

^a^ Correlation is significant at the 0.05 level (two-tailed). ^b^ Correlation is significant at the 0.01 level (two-tailed).

**Table 4:** Descriptive statistics, Cronbach’s alpha, and intraclass correlation coefficient (ICC) of the MEQ-M subscales and the MAAS.

Subscales	Cronbach’s Alpha	McDonald’s Omega	Mean ± SD	ICC (95% CI)
MEQ-M				
Environmental disinhibition	0.52	0.67	2.9 ± 0.39	0.320
Emotional response	0.62	0.80	3.1 ± 0.51	0.468
Taste awareness	0.70	0.71	2.4 ±0.52	0.256
Emotion awareness	0.62	0.64	2.7 ± 0.50	0.083
Portion disinhibition	0.54	0.58	2.5 ± 0.53	−0.173
External cues of food	0.27	NA	2.3 ± 0.59	−0.213
External cues of place	NA	NA	2.2 ± 0.85	−0.065
Summary score	0.64	0.44	2.6 ± 0.25	0.104
MAAS	0.88	0.89	4.57 ± 0.59	0.295

NA: Not available.

**Table 5:** Correlation between sociodemographic characteristics and the MEQ-M (Pearson’s r).

MEQ-M Subscales	Age	BMI	Gender	Education Level	Marital Status	Household Monthly Income
Environmental disinhibition	0.255 **	−0.207 *	0.169 *	−0.026	−0.015	0.082
Emotional response	0.266 **	−0.072	−0.017	0.048	0.018	0.127
Taste awareness	−0.077	0.115	0.125	0.017	0.053	−0.009
Emotion awareness	0.036	0.000	0.060	−0.060	0.006	−0.025
Portion disinhibition	−0.015	0.052	−0.007	0.048	0.038	−0.077
External cues of food	−0.071	0.066	0.137	0.149	−0.048	−0.043
External cues of place	−0.074	0.022	−0.009	0.000	−0.027	−0.104
Summary score	0.104	0.002	0.127	−0.017	0.007	−0.010

** Correlation is significant at the 0.01 level (two-tailed). * Correlation is significant at the 0.05 level (two-tailed).

## Data Availability

The data presented in this study is a part of an ongoing doctoral research of S.M.AB. Hence, we could not publicly release the data. However, it is available upon request from the corresponding author (Z.A.M.).

## Appendix A

**Table A1:** The Malay-translated Mindful Eating Questionnaire (MEQ).

Item	Question
1	I eat so quickly that I don’t taste what I’m eating.
	Saya makan dengan cepat sehingga saya tidak rasa apa yang saya makan.
2	When I eat at “all you can eat” buffets, I tend to overeat.
	Saya cenderung untuk terlebih makan apabila saya berada di jamuan buffet.
3	At a party where there is a lot of good food, I notice when it makes me want to eat more food than I should.
	Saya mempunyai keinginan untuk makan berlebihan sekiranya terdapat makanan yang lazat di sesebuah jamuan keramaian.
4	I recognize when food advertisements make me want to eat.
	Saya sedar iklan makanan akan membuatkan saya ingin makan.
5	When a restaurant portion is too large, I stop eating when I’m full.
	Apabila saiz porsi makanan di restoran/gerai terlalu besar, saya berhenti makan apabila sudah kenyang.
6	My thoughts tend to wander while I am eating.
	Fikiran saya cenderung melayang jauh/menerawang ketika sedang makan.
7	When I’m eating one of my favorite foods, I don’t recognize when I’ve had enough.
	Saya tidak sedar yang saya sudah kenyang apabila saya makan makanan yang saya gemari.
8	I notice that just going into a movie theatre makes me want to eat candy or popcorn.
	Saya ada keinginan untuk makan bertih jagung/makanan ringan apabila pergi menonton di pawagam.
9	If it doesn’t cost much more, I get the larger size food or drink regardless of how hungry I feel.
	Saya akan mengambil makanan/minuman bersaiz porsi besar tanpa mengira tahap kelaparan saya jika ia tidak melibatkan tambahan kos.
10	I notice when there are subtle flavors in the foods I eat.
	Saya dapat rasa/perasan jika terdapat sedikit perasa di dalam makanan saya.
11	If there are leftovers that I like, I take a second helping even though I’m full.
	Jika terdapat saki baki makanan yang saya gemari, saya akan makan lagi walaupun sudah kenyang.
12	When eating a pleasant meal, I notice if it makes me feel relaxed.
	Saya akan berasa tenang apabila saya makan makanan yang menyenangkan/menggembirakan.
13	I snack without noticing that I am eating.
	Saya mengudap tanpa saya sedari.
14	When I eat a big meal, I notice if it makes me feel heavy or sluggish.
	Saya akan merasa lesu selepas makan hidangan yang besar.
15	I appreciate the way my food looks on my plate.
	Saya menghargai rupa makanan yang dihidangkan di dalam pinggan saya.
16	I stop eating when I’m full even when eating something I love.
	Saya berhenti makan apabila sudah kenyang walaupun saya sedang makan makanan yang saya gemari.
17	When I’m feeling stressed at work, I’ll go find something to eat.
	Saya akan mencari makanan untuk dimakan sekiranya saya merasa tertekan di tempat kerja.
18	If there’s good food at a party, I’ll continue eating even after I’m full.
	Jika terdapat makanan yang lazat di jamuan keramaian, saya akan terus makan walaupun sudah kenyang.
19	When I’m sad I eat to feel better.
	Saya akan makan untuk menghilangkan kesedihan saya.
20	I notice when foods and drinks are too sweet.
	Saya dapat merasakan jika sesuatu makanan dan minuman itu terlalu manis.
21	Before I eat, I take a moment to appreciate the colors and smells of my food.
	Saya akan mengambil sedikit masa untuk menghargai warna dan bau makanan sebelum saya makan.
22	I taste every bite of food that I eat.
	Saya rasa setiap kunyahan makanan yang saya makan.
23	I recognize when I’m eating and I’m not hungry.
	Saya sedar apabila saya sedang makan dan tidak berasa lapar.
24	I notice when I’m eating from a dish of candy just because it’s there.
	Saya perasan saya makan sesuatu gula-gula/kerepek/makanan ringan hanya kerana ia berada berhampiran saya.
25	When I’m at a restaurant, I can tell when the portion I’ve been served is too large for me.
	Apabila saya berada di restoran/gerai makanan, saya dapat mengagak sekiranya saiz hidangannya terlalu besar bagi saya.
26	I notice when the food I eat affects my emotional state.
	Saya perasan jika sesuatu makanan itu memberi kesan kepada emosi saya.
27	I have trouble not eating ice cream, cookies, or chips if they’re around the house.
	Saya tidak dapat mengawal kemahuan untuk makan makanan ringan (spt. Aiskrim, biskut, kerepek) sekiranya makanan tersebut ada di rumah.
28	I think about things I need to do while I am eating.
	Saya memikirkan hal-hal lain yang saya perlu lakukan ketika sedang makan.
